# Correction: Dominant Negative Mutants of *Bacillus thuringiensis* Cry1Ab Toxin Function as Anti-Toxins: Demonstration of the Role of Oligomerization in Toxicity

**DOI:** 10.1371/annotation/0f267db9-6773-449d-b3c3-8f6c50e637ec

**Published:** 2013-02-28

**Authors:** Claudia Rodríguez-Almazán, Luis Enrique Zavala, Carlos Muñoz-Garay, Nuria Jiménez-Juárez, Sabino Pacheco, Luke Masson, Mario Soberón, Alejandra Bravo

There are corrections to Figure 2 and it's legend.

The correct figure is: 

**Figure:**
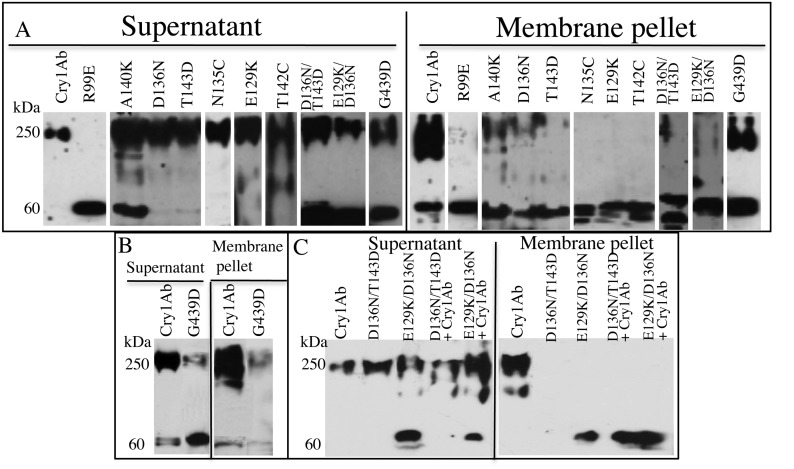


The correct legend is: Figure 2. Oligomerization of Cry1Ab proteins. Panel A, Cry1Ab and mutant protoxins were proteolytically activated with M sexta midgut proteases in the presence of SUV liposomes and scFv73 antibody. Membrane pellets were recovered by centrifugation and the toxin detected by Western blot using an anti-Cry1Ab antibody in the supernatant and in the membrane fraction. The oligomeric structure of 250-kDa of the wild type Cry1Ab toxin is observed inserted into the membrane pellet, in contrast with the helix α-4 mutants, that remain in the soluble fraction. The mutant R99E, located in helix α-3 did not form oligomeric structures. Panel B, Oligomerization of Cry1Ab and mutant G439D proteins performed as above but in the presence of the cadherin CR12 fragment instead of scFv73 antibody. Under these conditions the oligomerization of the Cry1Ab wild type is observed inserted into the membrane and oligomerization of G439D mutant was severely reduced. Panel C, Oligomerization of the mixtures of 1:1 Cry1Ab: Mutant proteins performed as in Panel A. The oligomer of double mutants or in the 1:1 mixture of Cry1Ab with the double mutants is observed in the soluble fraction. 

